# Use of handgrip strength measurement as an alternative for assessing chewing function in people with dementia

**DOI:** 10.1186/s12877-022-03452-2

**Published:** 2022-09-24

**Authors:** Julia Jockusch, Sebastian Hahnel, Ina Nitschke

**Affiliations:** 1 Department of Prosthodontics and Materials Science, Gerodontology Section, University of Leipzig, 04103 Leipzig, Germany; 2 University Research Priority Program, “Dynamics of Healthy Aging”, University of Zurich, 8050 Zurich, Switzerland; 3 Department of Prosthetic Dentistry, Regensburg University Medical Center, 93042 Regensburg, Germany; 4 Clinic of General, Special Care and Geriatric Dentistry, Center of Dental Medicine, University of Zurich, 8032 Zurich, Switzerland

**Keywords:** Cognitive impairment, Mini-Mental State Examination, Handgrip strength, Bite force, Chewing efficiency, Measurement, Dementia

## Abstract

**Background:**

Chewing ability and handgrip strength can be independent explanatory factors of physical fitness. The usability of measurement procedures for assessing chewing function in people with dementia seems to be limited. This study aimed to show an association between handgrip strength and chewing function to enable the use of handgrip strength measurement as an alternative for determining chewing parameters in people with dementia.

**Methods:**

The data analysed here are part of the OrBiD (Oral Health, Bite Force and Dementia) pilot study. A total of 120 participants were assigned to five evaluation groups based on their cognitive abilities using the Mini-Mental State Examination (MMSE). The MMSE groups in this data analysis were “no dementia” (noDem, MMSE 28–30), “mild cognitive impairment” (mCI, MMSE 25–27), and “mild dementia” (mDem, MMSE 18–24). Handgrip strength, maximum occlusal force, and chewing efficiency were measured.

**Results:**

The Mini-Mental State Examination scores among all participants (*n* = 71) resulted in a median of 27 and a range of 18–30. An association between maximum handgrip strength and the cognitive impairment of the participants was shown. Nevertheless, the use of handgrip strength measurement as an alternative for determining chewing function was not verified in this study.

**Conclusions:**

The feasibility and reliability of chewing function measurements in people with dementia should be investigated. Existing measurement procedures may need to be adapted or new assessments may need to be developed to be usable in people with dementia.

**Trial registration:**

ClinicalTrials.gov NCT03775772.

## Background

Chewing function can be described by the parameters of chewing ability, chewing efficiency, and bite force. Chewing ability (also known as masticatory ability) describes the subjective chewing ability of a person, which can be determined through questionnaires [1, 2]. Chewing efficiency (also known as masticatory efficiency) refers to objectively measurable chewing performance [3–8]. Bite force (also known as occlusal force or (maximum) occlusal force) is the physiologically possible force used to comminute a chewable item.

Chewing ability can be an independent predictor of physical fitness as measured through activities of daily living (ADL) [9]. Therefore, maintaining oral health, which may result in sufficient chewing ability, can improve activities of daily living [9]. It has also been observed that a low chewing ability is associated with lower ADL, lower cognitive functioning, depression, and food insufficiency in older people [10]. Thus, measurement of handgrip strength can be used to draw conclusions about health and physical performance [11].

Several studies have been conducted among community-dwelling older people with intact cognition, showing a relationship between handgrip strength and chewing function (i.e., subjective chewing ability, chewing efficiency, and bite force) [12–15]. It was also shown that participants with a higher body cell mass index (BCMI), higher handgrip strength, and more present teeth (i.e., teeth of the natural dentition (one’s own teeth) without taking dentures into account) had a significantly higher chance of achieving strong masseter muscle tension, tested by palpation of the muscle [14]. According to Moriya et al., a relationship might exist between self-assessed chewing ability and muscle strength (recorded as handgrip strength) of the body [16]. Furthermore, chewing efficiency is associated with handgrip strength in older community-dwelling people [15]. The maximum occlusal force (MOF), as a representation of bite force, is strong and independently associated with different measurements of physical performance in men, e.g., handgrip strength [13]. Additionally, different studies with community-dwelling older people showed a correlation between handgrip strength and MOF [17–19] and chewing function [17]. For people with dementia, at present, no studies have examined the relationship between handgrip strength and chewing function (i.e., chewing efficiency and bite force).

As handgrip strength is important for daily activities such as eating (i.e., picking up items) [20], it has been investigated in older people with dementia. Due to its ease in performing the measurement [11], isometric hand dynamometry with the Jamar® handheld dynamometer [21] is one of the most commonly used methods to measure muscle strength in people with dementia [22].

Performance-based strength and function measurements are reliable assessment methods in people with dementia. However, functional measurements appear to be more reliable than strength measurements [20]. Nevertheless, the reliability of the Jamar® handheld dynamometer is described as excellent in older people with dementia with a Mini-Mental State Examination (MMSE) of 10 to 28 [23], respectively, among those with borderline, mild, and moderate dementia [24].

Several studies have reported on the relationship between general cognition and chewing function in older people with dementia. (e.g., [10, 25]). One study concluded a causal relationship between cognition and mastication (i.e., chewing efficiency and chewing ability) found in animal and human experimental studies [26]. In a previous evaluation, the authors observed significant differences in chewing efficiencies and bite force between different degrees of cognitive impairment and dementia [27]. Another study reported on the association between chewing ability, tooth loss, and cognitive impairment, observing statistically significant higher odds of cognitive impairment using a self‐assessment of older people with dementia [28].

The disability and vulnerability of people with Alzheimer's disease and related dementias [29] who show reduced muscle mass [30] can result in difficulties/an inability to perform motor tasks that require muscular strength [29]. Therefore, it is not surprising that both bite force and handgrip strength [29] are associated with a loss in strength in people with dementia. Additionally, impaired motor skills in people with dementia appear to contribute to reduced chewing efficiency [31].

The use of self-reported information in older people with dementia is questionable due to its reliability [32]; the use of tests for measuring chewing function also have to be questioned. Weijenberg et al. reported a high dropout rate for the mixing ability test in older people with dementia to assess chewing efficiency [25]. Therefore, the usability of tests of chewing function in people with cognitive impairment or dementia seems to be limited. In the authors’ clinical practice, difficulties in conducting tests on bite force and chewing efficiency in people with advanced dementia are apparent due to problems in verbal comprehension, reduced motor skills, and compliance.

Iinuma et al. suggested that decreases in masticatory and skeletal muscle function due to age have mechanisms in common [13]. Perhaps this association can be utilised. In the clinical setting, being able to draw conclusions about chewing function from the measurement of handgrip strength would be helpful.

Because good chewing function is important for maintaining a balanced diet and may, in turn, positively influence body mass index and mortality, more attention should be paid to clinical measurement of chewing function in people with dementia. It has been shown that people without dementia up to mild dementia respond positively to masticatory muscle training [33]. Its effect on nutrition remains to be shown by future studies. Nevertheless, these studies and therapeutic approaches can also benefit people with dementia. However, as people with dementia are often unable to follow complex instructions due to cognitive and motor impairments, it would be of utmost importance for science and clinics to find alternative measurement methods that are feasible in people with dementia and that allow conclusions to be drawn about chewing function.

The aim of this study was therefore to show a possible association between handgrip strength and bite force, expressed as maximum occlusal force, or chewing efficiency as objective parameters of chewing function. This could enable the use of handgrip strength measurement as an alternative for determining bite force and chewing efficiency in people with cognitive impairment or dementia, in case these measurements are not suitable or feasible.

The authors, therefore, predicted a positive association between handgrip strength and maximum occlusal force as well as handgrip strength and chewing efficiency in people with and without cognitive impairment or dementia, intending to enable the use of handgrip strength measurements as an alternative for the measurement of chewing function.

## Methods

### Study design

Participants aged 60 years and older who were able to understand German and who were either cognitively healthy, cognitively impaired, or diagnosed with dementia were included in the study. People with all types of dementia could participate in the study. Participants with acute oral processes (pain, abscesses, etc.) that required emergency treatment were not included in the study until the emergency treatment was completed. Participants with physical limitations in the upper body due to musculoskeletal or neuromuscular conditions, e.g., paralysis of the arms, arthritis, post-stroke conditions affecting motor skills, facial nerve paralysis, etc., were excluded from the study. Individuals with additional disabilities which might have had an impact were also excluded (e.g., Trisomy 21, mental disabilities other than cognitive impairment/dementia, etc.).

The data analysed here are part of the OrBiD (Oral Health, Bite Force and Dementia) pilot study (ClinicalTrials.gov NCT03775772) [27, 33–36]. A total of 120 participants were assigned to five evaluation groups based on their cognitive abilities using the MMSE [37]. The MMSE groups in the OrBiD study were “no dementia” (noDem, MMSE 28–30), “mild cognitive impairment” (mCI, MMSE 25–27), “mild dementia” (mDem, MMSE 18–24), “moderate dementia” (modDem, MMSE 10–17), and “severe dementia” (sDem, MMSE ≤ 9). The allocation of the MMSE groups was based on the MMSE score categories described by Perneczky et al. [38]. Since the OrBiD study primarily tested the effects of interventions [33, 36] that can be integrated into everyday clinical practice, the authors opted for a slightly modified classification of dementia severity based on participants’ ability to receive therapy [39] and, associated with this, their ability to participate in assessments and measurement procedures within the study. The basis for setting the thresholds was an expert panel that indicated a range in the MMSE of 16–20 as the threshold for impairment of daily activities [35].

In this analysis, participants with moderate or severe dementia (MMSE ≤ 17) were excluded due to observed difficulties in implementing the study instructions when performing the tests for bite force and chewing efficiency. Therefore, the evaluation groups here are noDem, mCI, and mDem.

### Data collection

The recruitment process of the participants was adapted due to varied living locations and circumstances, depending on health, cognitive and organisational constraints. A detailed explanation of this process can be found in the literature [35]. Depending on the participants’ mobility, all participants of this analysis were evaluated by one examiner in a geriatric dental clinic or long-term care facilities. The examiner was one dentist with a specialisation in senior dentistry by the German Association of Gerodontology (DGAZ) and experience in senior dentistry and the handling of people with dementia in clinical settings. The study was conducted between September 2017 and June 2019.

### Measurements

Sociodemographic data such as age, sex, and living situation (community-dwelling vs. resident of a long-term care facility) were recorded.

The Mini Nutritional Assessment (MNA) [40] consists of a pre-assessment with 6 questions and the main anamnesis containing 12 additional questions with a maximum score of 30 points. A score of 17–23.5 points indicates the participant is at risk of malnutrition. A score of fewer than 17 points indicates poor nutritional status/malnutrition.

The MMSE [37], which tests verbal and non-verbal episodic memory, orientation in time and place, and visual constructive abilities (maximum score 30), was used to assign the participants to the evaluation groups. The MMSE was conducted by one dentist on all participants who did not have a medical diagnosis of dementia and did not provide information on their MMSE score in their requested medical reports.

**Fig. 1:**
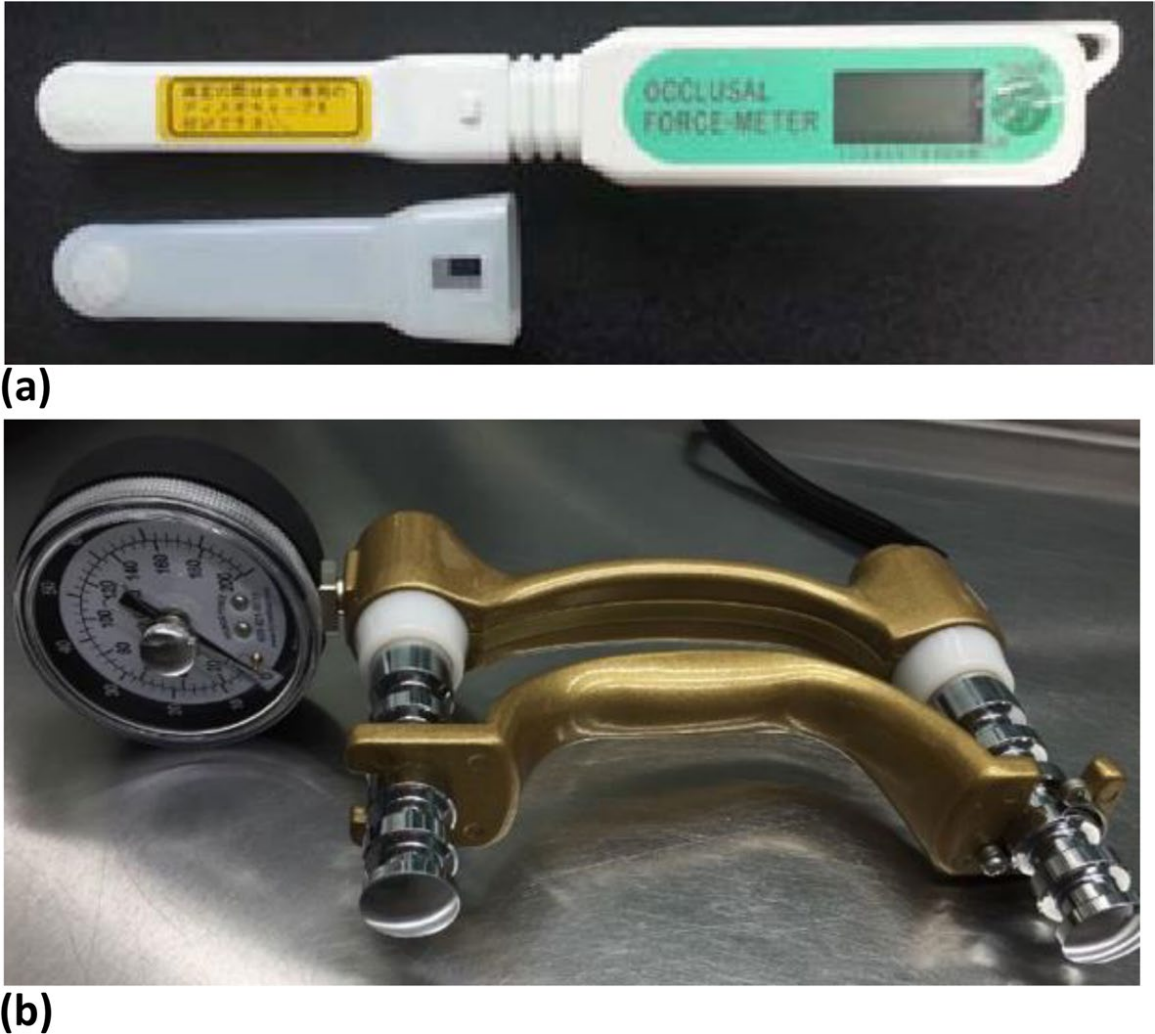
**a** Occlusal Force Meter GM 10® for measuring the bite force in Newton (N). **b** Jamar dynamometer

The measurement of bite force as maximum occlusal force (MOF, in Newton (N)) was performed with the participants in an unsupported, upright sitting position using a GM 10® occlusal force meter (Morita, Nagano Keiki, Higashimagome, Ohtaku, Tokyo, Japan) according to the procedure described in the literature [41] (Fig. 1a). Bite force measurement is performed in the region of the first molar (natural or artificial teeth replaced with bridge or dentures) by applying the maximum possible jaw closing force. When the first molar was missing and not artificially replaced, the measurement was performed at the area closest to the first molar (e.g., second molar/first premolar). Dentures were inserted if available, regardless of the type of denture (complete denture (no own natural teeth present) or partial dentures (partially own teeth present)). The measurement was recorded three times for each side of the jaw. Only the MOF achieved by the participants, which encompassed the maximum measurement of both sides of the jaw, was included in the analysis.

To determine chewing efficiency, the colour-mixing ability test was used as described by Schimmel et al. [8]. The investigator assessed the samples with the subjective visual assessment scale (five-step ordinal subjective assessment scale (SAS) according to Schimmel et al. (2007)) [8]. Additionally, an opto-electronical analysis with the free software ViewGum® (www.dhal.com) was carried out, calculating the hue value as a ratio of the unmixed fraction of the chewing gum to the total pixel number in a fixed-size template [42]. The variance of hue (VOH) is a measure of chewing efficiency. Adequate mixing of colours as an expression of good chewing efficiency will result in low VOH and vice versa [8, 42].

Handgrip strength measurement was carried out with the Jamar® dynamometer, which has been described as a valid measuring instrument [21, 43]. The participant sits upright on a chair with back support and fixed armrests. Participants are asked to rest their forearms on the arms of the chair and keep their feet flat on the floor. The measuring device is held vertically, with the upper arm suspended in a relaxed position and the forearm angled at 90 degrees. The wrist is slightly bent (about 30 degrees to the forearm). The examiner supports the measuring device from below and instructs the participant to squeeze the grip handle as hard as possible (Fig. 1b).

The participant’s hand dominance was recorded but is not analysed here. The measurement of handgrip strength was recorded three times for each hand. The maximum value (in kilogram, kg) of both sides achieved by the participant was included in the analysis as maximum handgrip strength.

The measurement was carried out according to the Southampton protocol [44], but without asking the participant to take off their shoes, as this was not possible for participants in need of care.

### Statistical analysis

Since this study was a pilot study, a power calculation was not performed due to the missing description of endpoints in the literature. The sample size was estimated based on similarly designed studies [45, 46].

All statistical analyses and plots were computed with the statistical software R [47], including the packages tidyverse [48], mice [49] and missForest [50], or SPSS 23.0 for descriptive statistics [51]. Missing values were identified and imputed using the missForest algorithm [50].

A descriptive analysis was performed for all the measured parameters (frequencies, median, and range, mean and SD). Graphical tools were used where appropriate to show potential associations between parameters. The Kruskal–Wallis test for independent samples was used to examine whether the independent samples (evaluation groups) differed statistically significantly for the individual parameters examined. The level of significance (α) was set at p < 0.05.

### Ethical considerations

The data of this analysis are part of a controlled clinical pilot study with interventions and stratified randomisation, called the “Oral Health, Bite Force and Dementia” (OrBiD) study (clinicaltrials.org number: NCT03775772).

The study was approved by the competent Cantonal Ethics Committee (CEC) of Zurich (KEK-ZH 2017–00,363). All participants or their legal representatives gave informed consent.

## Results

### Study population

A total of 71 participants (age: mean 78.3 years ± 9.3 years, median 79 years (range 61–95 years), 66.2% female) were included in the analysis. A description of the participants of this analysis regarding their dental and denture status stratified by evaluation group noDem, mCI, and mDem (e.g., number of supporting zones, number of natural teeth, presence, type, and quality of denture) can be found in the literature [27]. Table 1 provides an overview of additional parameters of the participants.

**Table 1:** Study parameters stratified by evaluation group

Study parameters	Total *n* = 71	noDem *n* = 24	mCI *n* = 24	mDem *n* = 23	*p*
**Age** [years]					
Mean ± SD	78.3 ± 9.3	74.1 ± 8.3	78.8 ± 10.3	82.2 ± 7.5	**0.01**
Median (range)	79 (61–95)	75 (62–92)	81 (61–95)	85 (65–95)	
**Sex** [n/%]					
Male	24/33.8	11/45.8	8/33.3	5/21.7	0.222
Female	47/66.2	13/54.2	16/66.7	18/78.3	
**Living situation** [n/%]					
Long-term care facility	30/42.3	1/4.2	10/41.7	19 /82.6	** < 0.001**
Community-dwelling	41/57.7	23/95.8	14/58.3	4/17.4	
**Mini-Mental State Examination** (MMSE)					
Mean ± SD	25.5 ± 3.8	29.1 ± 0.7	26.5 ± 0.7	20.8 ± 2.5	** < 0.001**
Median (range)	27 (18–30)	29 (28–30)	27 (25–27)	21 (18–24)	
**Chewing efficiency**					
*Variance of hue (VOH)*					
Mean ± SD	0.3 ± 0.2	0.3 ± 0.2	0.2 ± 0.2	0.3 ± 0.2	0.522
*Subjective assessment scale (SAS)* [n/%]					
SA1	10/14.1	3/12.5	3/12.5	4/17.4	0.507
SA2	19/26.8	8/33.3	4/16.7	7/30.4	
SA3	30/42.3	6/25.0	14/58.3	10/43.5	
SA4	7/9.9	5/20.8	1/4.2	1/4.3	
SA5	5/7.0	2/8.3	2/8.3	1/4.3	
**Maximum occlusal force** (MOF) [kN]					0.282
Mean ± SD	0.2 ± 0.2	0.1 ± 0.2	0.2 ± 0.2	0.1 ± 0.1	
**Maximum handgrip strength** [kg]					
Mean ± SD					**0.003**
All participants	21.2 ± 11.1	26.4 ± 10.9	20.7 ± 10.3	16.1 ± 9.8	
Males	31.8 ± 9.7	33.9 ± 11.6	32.3 ± 7.8	26.4 ± 7.0	
Females	15.6 ± 7.0	20 ± 4.5	14.9 ± 5.5	13.2 ± 8.6	
**Mini Nutritional Assessment** [n/%]	*n* = 32	*n* = 7	*n* = 13	*n* = 12	
Well-nourished	0/0	0/0	0/0	0/0	**0.002**
Risk of malnutrition	12/37.5	2/28.6	4/30.8	6/50.0	
Malnutrition	20/62.5	5/71.4	9/69.2	6/50.0	

Bold values in column p indicate statistical significance with a significance level of *p* < 0.05, *p* = Kruskal–Wallis

*noDem* no dementia, *mCI* mild cognitive impairment, *mDem* mild dementia

### Chewing efficiency, bite force (expressed as maximum occlusal force), and maximum handgrip strength

**Fig. 2:**
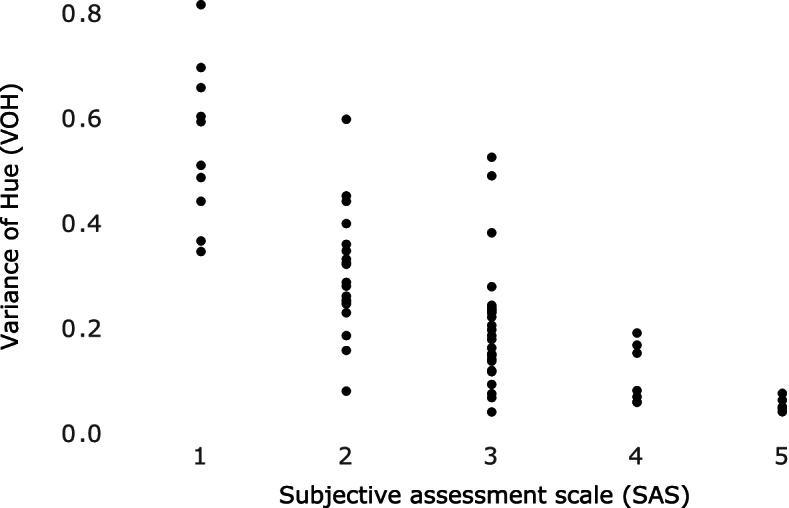
Dot plot showing the association between the variance of hue (VOH) and the subjective assessment scale (SAS) in people with and without cognitive impairment and/or dementia. (SAS: 5-step ordinal subjective assessment scale. According to Schimmel et al. 2007 [8], SA1—chewing gum not mixed, impressions of cusps or folded once; SA2—large parts of chewing gum unmixed; SA3—bolus slightly mixed, but bits of unmixed original colour; SA4—bolus well mixed, but colour not uniform; SA5—bolus perfectly mixed with uniform colour. A degree of mixing of SA1 or SA2 can be interpreted as difficulties in enjoying normal meals.)

An association between the VOH and SAS was shown in the analysis, confirming the usability of the colour-mixing ability test according to Schimmel et al. [8] in participants with and without cognitive impairment or dementia. Low VOH values and a high score on the SAS indicate good chewing efficiency (Fig. 2).

**Fig. 3:**
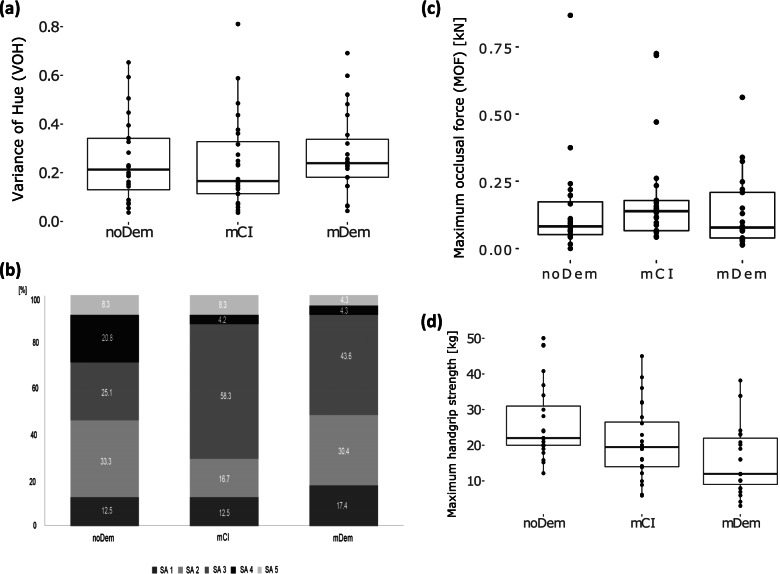
Chewing efficiency, maximum occlusal force, and maximum handgrip strength are stratified by evaluation group (noDem—no dementia; mCI—mild cognitive impairment; mDem—mild dementia). **a** Chewing efficiency and variance of hue (VOH) (lower values indicate adequate mixing of colours, representing a good chewing efficiency). **b** Chewing efficiency and subjective assessment scale (SAS) (SA1—chewing gum not mixed, impressions of cusps or folded once; SA2—large parts of chewing gum unmixed; SA3—bolus slightly mixed, but bits of unmixed original colour; SA4—bolus well mixed, but colour not uniform; SA5—bolus perfectly mixed with uniform colour). **c** Maximum occlusal force (MOF, in kN). **d** Maximum handgrip strength (in kg)

The VOH as an expression of chewing efficiency showed only slight—clinically irrelevant—differences between people with mCI and mDem. Overall, no statistical difference in VOH between the evaluation groups was observed (Fig. 3a, Table 1).

The SAS showed almost no differences in the categories SA1 and SA2 (difficulties in enjoying meals [8]) between the evaluation groups noDem and mDem. Participants with mCI demonstrated these degrees of impaired chewing efficiency less often. The opposite of the scale revealed three times more participants with noDem (SA4/SA5: *n* = 7, 29.1%) with a good or very good chewing efficiency in terms of the SA categories SA4 and SA5, compared with the evaluation groups mCI (SA4/SA5: *n* = 3, 12.5%) or mDem (SA4/SA5: *n* = 2, 8.6%). No statistical differences were observed between the evaluation groups for the SAS (Fig. 3b, Table 1) and MOF (Fig. 3c, Table 1).

A statistically significant difference (*p* = 0.003, Kruskal–Wallis) in maximum handgrip strength was recorded between the participants with noDem and mDem. Overall, a tendency was observed to show a reduction in maximum handgrip strength with the increase in cognitive impairment (Fig. 3d, Table 1).

### Association between handgrip strength and chewing function

**Fig. 4:**
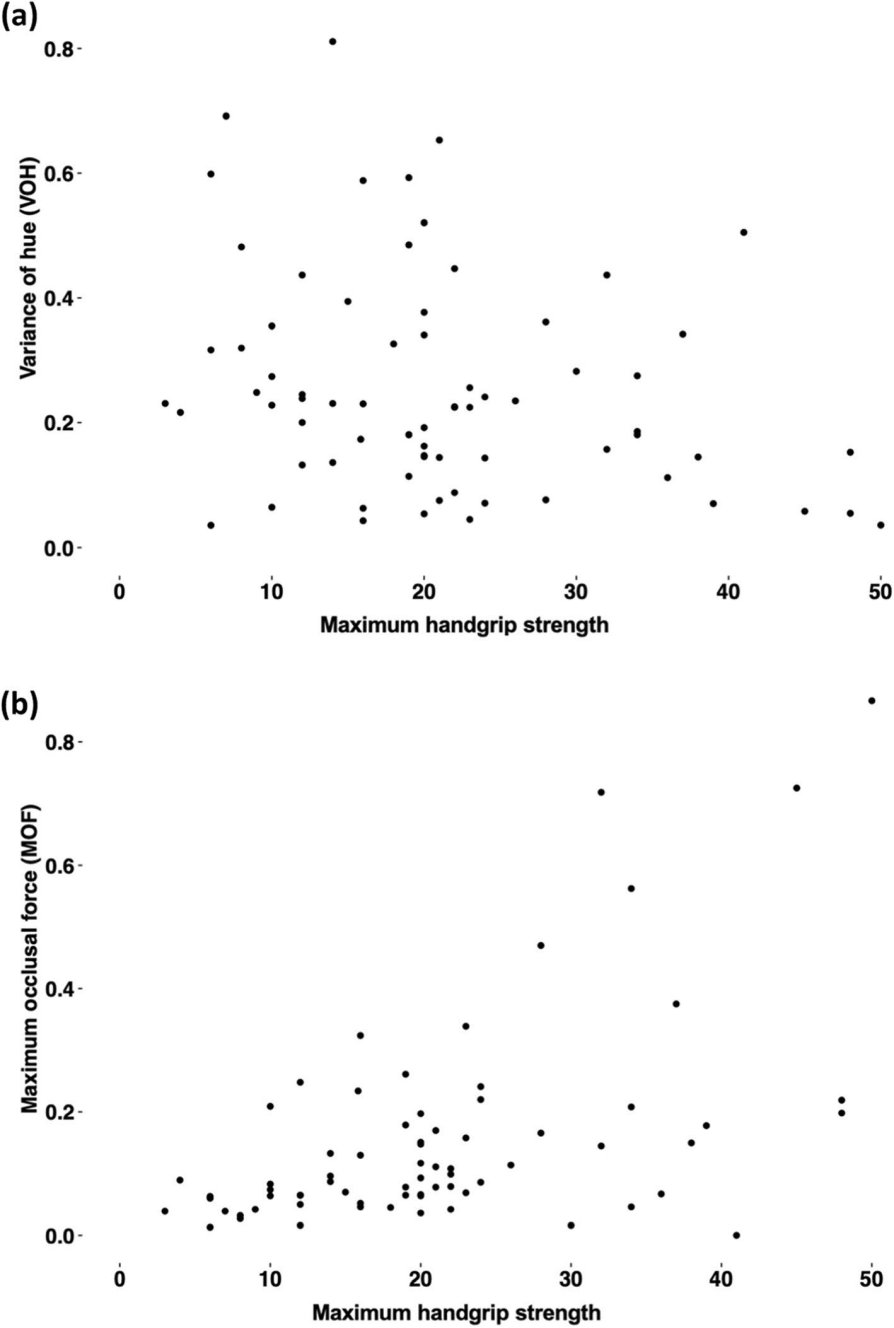
Association between maximum handgrip strength and (**a**) Chewing efficiency (Variance of hue (VOH)) and (**b**) Maximum occlusal force (MOF)

Despite the observed association between handgrip strength and cognitive function (Fig. 3d), no association between maximum handgrip strength and the parameters of chewing function (i.e., VOH and MOF) was found in this study (Figs. 4 a and b).

Additionally, no association between maximum handgrip strength and chewing efficiency, visually evaluated with the subjective assessment scale (SAS), was found (Table 2).

**Table 2:** Association between maximum handgrip strength and the subjective assessment scale (SAS)

	**Maximum handgrip strength** [kg]		
**Subjective Assessment Scale** (SAS)	**Mean ± SD**	**Median**	**IQR**
SA1	17.3 ± 10.8	15	11.8
SA2	22.3 ± 11.3	22	17.5
SA3	18.8 ± 9.0	19	9.5
SA4	25.7 ± 12.4	20	11.0
SA5	31.6 ± 14.9	24	22.0

*IQR* Interquartile range, *SAS* Subjective assessment scale: SA1—chewing gum not mixed, impressions of cusps or folded once; SA2—large parts of chewing gum unmixed; SA3—bolus slightly mixed, but bits of unmixed original colour; SA4—bolus well mixed, but colour not uniform; SA5—bolus perfectly mixed with uniform colour

## Discussion

In this study, an association between VOH and the visual SAS was shown, confirming the usability of the colour-mixing ability test in people with and without cognitive impairment or dementia. A statistically significant difference was also observed between the participants with noDem and mDem for maximum handgrip strength. Overall, a trend was observed showing a reduction in maximum handgrip strength with an increase in cognitive impairment. Nevertheless, associations between handgrip strength and the parameters of chewing function (i.e., chewing efficiency (VOH, SAS) and MOF) could not be proven in this study. The authors’ hypothesis must therefore be rejected.

The study has several limitations. The participants differed not only concerning the MMSE, but also in terms of the living situation (i.e., long-term care facilities and community-dwelling settings). The extent to which place of residence and general living situation can influence the enjoyment of food or the ability to chew was not considered in this study. An older person’s healthy psychological situation may contribute to a more sustained use of chewing muscles; however, this would require specific investigation.

Differences in age and gender (higher age or higher proportion of women with increasing dementia) can be explained by gender differences in demography.

Other oral factors, such as the number of teeth, the number of occlusal surfaces [52–56], and the type of denture [6, 57–60], can be expected to influence the outcome of masticatory function. They thus have an impact on the observed association between handgrip strength and chewing function as a function of cognitive ability. The authors’ original aim was to measure handgrip strength as an alternative to determine the maximum occlusal force and masticatory efficiency in people with cognitive impairment or dementia, in case these measurement protocols are infeasible. The analysis of this study has only been conducted in this context.

In the literature, a significant relationship between occlusal force and cognition is described after controlling for possible explanatory factors, including handgrip strength as an indicator of general muscle strength, demonstrating oral function independence [61]. The cognitive status of the participants in this study was reported with a mean MoCA-J (Montreal Cognitive Assessment, Japanese version) score of 22.5, which corresponds [62] approximately to the mean MMSE score of 25.5 of all the participants in this analysis. Nevertheless, a comparison is difficult because, in the study by Takeshita et al., participants were grouped based on age rather than cognitive function, and the number of participants with mild cognitive impairment or mild dementia was not recorded [61]. Therefore, it should be investigated further whether independence in oral function should be given in participants with impaired cognition and, in particular, severe dementia. However, this was not the aim of the present study.

Furthermore, the discriminating power of the MMSE classification chosen in this analysis should be critically questioned. A different selection of cut-off values in the MMSE could possibly influence the outcome variables. An MMSE cut-off score of 23 is described as an ideal value to detect dementia with the MMSE instrument. However, the cut-off is dependent on the level of education [63, 64]. Additionally, the use of the MMSE in general must be questioned. For example, the MoCA test is known to meet the criteria for screening tests for mild cognitive impairment better than the MMSE [65]. Nevertheless, the authors decided to use the MMSE in the study because it is commonly used around the world, is time efficient, and was often available in the participants’ medical records.

In the present analysis, only data from participants who were able to understand and independently carry out the tests were included in the analysis. Participants whose verbal or manual skills in dealing with the test procedures raised doubts about their ability to perform the tasks were excluded from the analysis (*n* = 1 in the mDem group). This was the only way to ensure that the results were not influenced by inability to perform a test. The authors have deliberately refrained from using mean values for the analysis of data for both occlusal force and handgrip strength, despite the measurements being repeatedly performed for each variable. This was due to the fact that the investigator repeatedly noticed during the clinical trial that handgrip strength values were highest mainly during the first measurements. This could have been caused by rapid fatigue in the older participants. In contrast, the mean values of occlusal force were often highest only during the second measurement. This might be due to the fact that the participants had only “tried out” the test instrument the first time. Some participants expressed fears that this procedure may cause pain or damage to teeth and dentures. This study was not able to show a decline in maximum occlusal force as a function of the early stages of cognitive decline in an older population, as described in the literature [61]. A causal relationship between cognition and MOF (here, in community-dwelling older people [19, 61, 66]) and chewing efficiency, which has previously been shown in animal and human experimental studies [26], has been reported in the literature. This has not been confirmed in the present study. To our knowledge, this is the first study to assess an association between chewing efficiency and handgrip strength in a population with cognitive impairment or mild dementia. Therefore, the current findings cannot be directly compared to other studies. The reason for this may be publication bias: studies with positive results may be more represented in the literature [67].

Handgrip strength measurements are dependent on age [68], sex, and ethnicity [69]. Age and gender-adapted norm values are additionally influenced by many factors such as height, weight, comorbidities, profession [70], and the nutritional status [71] of the participants. Therefore, values comparable to this study are hard to find in the literature because of different confounding variables in other studies. In this study, statistically significant differences in handgrip strength values as a function of the cognition of the participants have been observed. This corresponds to the literature. For example, Su et al. showed an association between a stronger handgrip strength and better performance in terms of cognitive function [72]. Conversely, Esteban-Cornejo et al. observed an association between a lower handgrip strength and a higher risk of all-cause dementia incidence and mortality [73]. The clinical significance of these differences was also investigated by McGrath et al., who demonstrated that every 5 kg higher handgrip strength was associated with “0.97 lower odds for both future cognitive impairment and worse cognitive impairment” [74].

Statistically, the number of participants was considered high enough to be able to answer the authors’ question. A significantly higher number of cases can be assumed to have no further influence on the examined associations, since the medians of the examined parameters in the evaluation groups were close to each other.

The results of this study may have arisen due to various circumstances. It is therefore important to consider the possibility that a different statistical analysis or the use of cut-off values for handgrip strength adjusted for gender and body mass index [75, 76] may have led to different results. As data may be analysed in different ways, it is important that the process is pre-specified in the study protocol. As a result, we followed our protocol and considered the correct approach from a scientific perspective.

In addition, study participants who were unable to perform the measurements due to cognitive impairment or dementia were excluded. The inclusion of a larger number of study participants with dementia who were able to perform the measurements might have been beneficial for the validity of the study.

The authors assume that, since no association between handgrip strength and parameters of chewing function has been observed in this study, measurement of handgrip strength with the Jamar dynamometer does not appear to be a possible alternative to provide inferences about the chewing function of people with dementia.

Although various associations between chewing function and dementia have been described in the literature (e.g., [10, 25, 26, 28]), the method of carrying out the tests (e.g., mixing ability test [25]) and their reliability in people with dementia should be examined further. The main question arises as to how reliable the testing of chewing efficiency and MOF is in people with cognitive impairment and dementia. In the current study, only one test procedure each was used to evaluate MOF and chewing efficiency. Future studies should therefore investigate whether other test procedures for chewing efficiency (e.g., sieve method [3, 4], computer-aided particle analysis [5], degree of carrot comminution [6], chewing gum [77, 78], image analysis [79]) or for measuring bite force [80], with regard to performance, the compliance of the participants tested and reliability, are able to obtain more reliable results. Even the development of new diagnostic tests for chewing efficiency and occlusal force for people with dementia should be considered. Trautwein et al. conclude there is a “need for tailoring motor assessments” to people with cognitive impairment or dementia. In addition, the need for standardised assessment procedures has been emphasised [81].

An adaptation and analysis of the available test procedures is required in order to include people with cognitive impairment and dementia in research and to address age and cognitive impairment discrimination in studies. Additionally, feasible alternative measurements of muscle function, strength, and mass as alternatives for handgrip strength measurement that may be carried out by people with cognitive impairment or dementia should be considered in further studies. The European Working Group on Sarcopenia in Older People has made suggestions regarding measuring methods used in research or clinical practice to evaluate the parameters of sarcopenia [82]. Concerning muscle mass measurements, computed tomography (CT), and magnetic resonance imaging (MRI) are described as the gold standard in the estimation of muscle mass but are mostly relevant for research purposes due to the radiation exposure patients are subjected to, as well as their high cost [82, 83]. An alternative is dual energy X-ray absorptiometry (DXA), which is used in clinical practice and research settings [83]. Bioimpedance analysis (BIA) may represent a well-studied [84], reproducible and easy-to-use portable alternative for DXA [82] in older subjects [85–87]. However, anthropometric measurements, which are easy to obtain in clinical practice, are not recommended for the diagnosis of sarcopenia [82]. Regarding muscle strength, there are fewer measuring methods available. Handgrip strength seems to be a simple and reliable method to measure muscle strength. Additionally, it is easier to carry out than measurement of the lower arm or leg strength [82]. Alternatives may include the measurement of peak expiratory flow or the measurement of knee flexion/extension, neither of which is recommended in clinical practice [82]. Since the main problem in this study concerned the measurement of chewing function or maximum occlusal force, the measurement of handgrip strength should be retained as an assessment method. Regarding physical function, the Short Physical Performance Battery (SPPB) can be used as a standard measure both in clinical practice and research [82, 88]. Next to the SPPB, the measurement of gait speed [88], and the timed get-up-and-go test may be helpful in clinical practice to define patients’ physical performance [82].

An ideal alternative for measuring hand force or even the parameters of a patient’s chewing point would need to meet various requirements. These include, among other things, the possibility of enabling the measurements in everyday life or their verification through observations or arbitrary reflexes. Furthermore, it would be purposeful if the measurements could be obtained without instructions, requirements, or complex devices and still be reliable and replicable at any time.

The authors assume that people with dementia could also benefit from an improvement in chewing function, as its associations with quality of life [9, 89], activities of daily living [9, 10], and nutritional status [89] are evident. Measurements of nutrition and quality of life should therefore be the subject of future clinical trials. This article provides an initial starting point to overcoming existing limitations in studies with people with cognitive impairment and dementia by identifying future research questions. The use of sequential approaches in the development of test procedures, characterised by a gradual increase in complexity from simple to more difficult tasks, is recommended. Furthermore, tests used for motor skills assessment should have sufficient relative reliability for people with dementia. In their implementation, instructions should be adapted to the cognitive capacity of the participants. External guidance and assistance should also be allowed in test procedures [81].

## Conclusions

The use of handgrip strength measurement as an alternative for determining the maximum occlusal force and chewing efficiency, tested with the GM 10® occlusal force meter and the colour-mixing ability test in people with cognitive impairment or dementia, has not been validated in this study. Further evaluations of chewing function are needed in terms of its feasibility and reliability in people with cognitive impairment and dementia. Adapting existing test procedures or developing new procedures that are easy to use for people with dementia may also be necessary.

## Data Availability

The datasets generated and/or analysed during the current study are not publicly available due to the ethics approval guidelines but are available from the corresponding author upon reasonable request.

## References

[1] Agerberg G, Carlsson GE (1981). Chewing Ability in Relation to Dental and General Health: Analyses of Data Obtained From a Questionnaire. Acta Odontol Scand.

[2] Boretti G, Bickel M, Geering AH (1995). A Review of Masticatory Ability and Efficiency. J Prosthet Dent.

[3] Fontijn-Tekamp FA, Slagter AP, Van Der Bilt A, Van ’T Hof MA, Witter DJ, Kalk W, Jansen JA (2000). Biting and Chewing in Overdentures, Full Dentures, and Natural Dentitions. J Dent Res.

[4] Miyawaki S, Ohkochi N, Kawakami T, Sugimura M (2001). Changes in Masticatory Muscle Activity According to Food Size in Experimental Human Mastication. J Oral Rehabil.

[5] Mowlana F, Heath MR, Bilt A, Glas HW (1994). Assessment of Chewing Efficiency: A Comparison of Particle Size Distribution Determined Using Optical Scanning and Sieving of Almonds. J Oral Rehabil.

[6] Wöstmann B, Wickop H, Kolb G, Ferger P. Zahnärztlich geriatrisches Assessment zur objektiven Einschätzung der zahnärztlich prothetischen Versorgung und des Ernährungszustandes älterer Patienten. Geriat Forsch. 1997;7:112–3.

[7] Prinz JF (1999). Quantitative Evaluation of the Effect of Bolus Size and Number of Chewing Strokes on the Intra-Oral Mixing of a Two-Colour Chewing Gum. J Oral Rehabil.

[8] Schimmel M, Christou P, Herrmann F, Müller F (2007). A Two-Colour Chewing Gum Test for Masticatory Efficiency: Development of Different Assessment Methods. J Oral Rehabil.

[9] Takata Y, Ansai T, Awano S, Hamasaki T, Yoshitake Y, Kimura Y, Sonoki K, Wakisaka M, Fukuhara M, Takehara T (2004). Relationship of Physical Fitness to Chewing in an 80-Year-Old Population. Oral Dis.

[10] Kimura Y, Ogawa H, Yoshihara A, Yamaga T, Takiguchi T, Wada T, Sakamoto R, Ishimoto Y, Fukutomi E, Chen W (2013). Evaluation of Chewing Ability and Its Relationship with Activities of Daily Living, Depression, Cognitive Status and Food Intake in the Community-Dwelling Elderly: Evaluation of Chewing Ability with CGA. Geriatr Gerontol Int.

[11] Shin H-S (2019). Handgrip Strength and the Number of Teeth among Korean Population. J Periodontol.

[12] Moriya S, Tei K, Yamazaki Y, Hata H, Shinkai S, Yoshida H, Muramatsu M, Kitagawa Y, Inoue N, Yamada H (2011). Relationships between Perceived Chewing Ability and Muscle Strength of the Body among the Elderly: CHEWING ABILITY AND MUSCLE STRENGTH. J Oral Rehabil.

[13] Iinuma T, Arai Y, Fukumoto M, Takayama M, Abe Y, Asakura K, Nishiwaki Y, Takebayashi T, Iwase T, Komiyama K (2012). Maximum Occlusal Force and Physical Performance in the Oldest Old: The Tokyo Oldest Old Survey on Total Health. J Am Geriatr Soc.

[14] Gaszynski T, Gaszynska E, Godala M, Szatko F (2014). Masseter Muscle Tension, Chewing Ability, and Selected Parameters of Physical Fitness in Elderly Care Home Residents in Lodz. Poland. CIA.

[15] Izuno H, Hori K, Sawada M, Fukuda M, Hatayama C, Ito K, Nomura Y, Inoue M (2016). Physical Fitness and Oral Function in Community-Dwelling Older People: A Pilot Study. Gerodontology.

[16] Moriya S, Muramatsu T, Tei K, Nakamura K, Muramatsu M, Notani K, Inoue N (2009). Relationships between Oral Conditions and Physical Performance in a Rural Elderly Population in Japan. Int Dent J.

[17] Mihara Y, Matsuda K, Ikebe K, Hatta K, Fukutake M, Enoki K, Ogawa T, Takeshita H, Inomata C, Gondo Y (2018). Association of Handgrip Strength with Various Oral Functions in 82- to 84-Year-Old Community-Dwelling Japanese. Gerodontology.

[18] Eto M, Miyauchi S (2018). Relationship between Occlusal Force and Falls among Community-Dwelling Elderly in Japan: A Cross-Sectional Correlative Study. BMC Geriatr.

[19] Iinuma T, Arai Y, Takayama M, Abe Y, Ito T, Kondo Y, Hirose N, Gionhaku N (2016). Association between Maximum Occlusal Force and 3-Year All-Cause Mortality in Community-Dwelling Elderly People. BMC Oral Health.

[20] Thomas VS, Hageman PA (2002). A Preliminary Study on the Reliability of Physical Performance Measures in Older Day-Care Center Clients With Dementia. Int Psychogeriatr.

[21] Mathiowetz V, Weber K, Volland G, Kashman N (1984). Reliability and Validity of Grip and Pinch Strength Evaluations. The Journal of Hand Surgery.

[22] Shechtman O, Mann WC, Justiss MD, Tomita M (2004). Grip Strength in the Frail Elderly. Am J Phys Med Rehabil.

[23] Blankevoort CG, van Heuvelen MJG, Scherder EJA (2013). Reliability of Six Physical Performance Tests in Older People With Dementia. Phys Ther.

[24] Alencar MA, Dias JMD, Figueiredo LC, Dias RC (2012). Handgrip Strength in Elderly with Dementia: Study of Reliability. Rev Bras Fisioter.

[25] Weijenberg RAF, Lobbezoo F, Visscher CM, Scherder EJA (2015). Oral Mixing Ability and Cognition in Elderly Persons with Dementia: A Cross-Sectional Study. J Oral Rehabil.

[26] Weijenberg RAF, Scherder EJA, Lobbezoo F (2011). Mastication for the Mind—The Relationship between Mastication and Cognition in Ageing and Dementia. Neurosci Biobehav Rev.

[27] Jockusch J, Hopfenmüller W, Nitschke I (2021). Chewing Function and Related Parameters as a Function of the Degree of Dementia: Is There a Link between the Brain and the Mouth?. J Oral Rehabil.

[28] Lexomboon D, Trulsson M, Wårdh I, Parker MG (2012). Chewing Ability and Tooth Loss: Association with Cognitive Impairment in an Elderly Population Study. J Am Geriatr Soc.

[29] Rogers SD, Jarrot SE (2008). Cognitive Impairment and Effects on Upper Body Strength of Adults with Dementia. J Aging Phys Act.

[30] Nourhashémi F, Andrieu S, Gillette-Guyonnet S, Reynish E, Albarède JL, Grandjean H, Vellas B (2002). Is There a Relationship Between Fat-Free Soft Tissue Mass and Low Cognitive Function? Results From a Study of 7,105 Women. J Am Geriatr Soc.

[31] Elsig F, Schimmel M, Duvernay E, Giannelli SV, Graf CE, Carlier S, Herrmann FR, Michel J-P, Gold G, Zekry D (2015). Tooth Loss, Chewing Efficiency and Cognitive Impairment in Geriatric Patients. Gerodontology.

[32] Zuluaga DJM, Montoya JAG, Contreras CI, Herrera RR (2012). Association between Oral Health, Cognitive Impairment and Oral Health-Related Quality of Life: Cognition and OH-QoL. Gerodontology.

[33] Jockusch J, Hahnel S, Sobotta BBAJ, Nitschke I (2022). The Effect of a Masticatory Muscle Training Program on Chewing Efficiency and Bite Force in People with Dementia. IJERPH.

[34] Jockusch J, Hopfenmüller W, Nitschke I (2021). Influence of Cognitive Impairment and Dementia on Oral Health and the Utilization of Dental Services: Findings of the Oral Health, Bite Force and Dementia Study (OrBiD). BMC Oral Health.

[35] Jockusch J, Wiedemeier D, Nitschke I (2022). The OrBiD (Oral Health, Bite Force and Dementia) Pilot Study: A Study Protocol for New Approaches to Masticatory Muscle Training and Efficient Recruitment for Longitudinal Studies in People with Dementia. Int J Environ Res Public Health.

[36] Jockusch J, Nitschke S, Hopfenmüller W, Schierz O, Hahnel S, Nitschke I (2022). Impact of an Oral Hygiene Intervention in People with and without Dementia on Oral Health Parameters-Results from the Oral Health, Bite Force, and Dementia (OrBiD) Pilot Study. J Clin Med.

[37] Folstein MF, Folstein SE, McHugh PR (1975). “Mini-Mental State”. A Practical Method for Grading the Cognitive State of Patients for the Clinician. J Psychiatr Res.

[38] Perneczky R, Wagenpfeil S, Komossa K, Grimmer T, Diehl J, Kurz A (2006). Mapping Scores onto Stages: Mini-Mental State Examination and Clinical Dementia Rating. Am J Geriatr Psychiatry.

[39] Nitschke I, Hopfenmüller W (1996). Die Zahnmedizinische Versorgung Älterer Menschen. [Dental Care for Older People.]. Die Berliner Altersstudie. [The Berlin Aging Study.].

[40] Rubenstein LZ, Harker JO, Salva A, Guigoz Y, Vellas B (2001). Screening for Undernutrition in Geriatric Practice: Developing the Short-Form Mini-Nutritional Assessment (MNA-SF). J Gerontol A Biol Sci Med Sci.

[41] Varga S, Spalj S, Lapter Varga M, Anic Milosevic S, Mestrovic S, Slaj M (2011). Maximum Voluntary Molar Bite Force in Subjects with Normal Occlusion. Eur J Orthod.

[42] Schimmel M, Christou P, Miyazaki H, Halazonetis D, Herrmann FR, Müller F (2015). A Novel Colourimetric Technique to Assess Chewing Function Using Two-Coloured Specimens: Validation and Application. J Dent.

[43] Abizanda P, Navarro JL, García-Tomás MI, López-Jiménez E, Martínez-Sánchez E, Paterna G (2012). Validity and Usefulness of Hand-Held Dynamometry for Measuring Muscle Strength in Community-Dwelling Older Persons. Arch Gerontol Geriatr.

[44] NIHR Southampton Biomedical Research Centre. Procedure for Measuring Handgrip Strength Using the Jamar Dynamometer. Version 2. 2014. https://www.uhs.nhs.uk/Media/Southampton-Clinical-Research/Procedures/BRCProcedures/Procedure-for-measuring-gripstrength-using-the-JAMAR-dynamometer.pdf. Accessed 20 Sept 2022.

[45] Teare MD, Dimairo M, Shephard N, Hayman A, Whitehead A, Walters SJ (2014). Sample Size Requirements to Estimate Key Design Parameters from External Pilot Randomised Controlled Trials: A Simulation Study. Trials.

[46] Malekmahmoodi M, Shamsi M, Roozbahani N, Moradzadeh R (2020). A Randomized Controlled Trial of an Educational Intervention to Promote Oral and Dental Health of Patients with Type 2 Diabetes Mellitus. BMC Public Health.

[47] R Core Team. R: A Language and Environment for Statistical Computing. 2013

[48] Wickham H. Tidyverse: Easily Install and Load the ‘Tidyverse’. R Package Version 1.2.1. 2017.

[49] Van Buuren S, Groothuis-Oudshoorn K. mice: Multivariate imputation by chained equations in R. J Stat Softw. 2011;45:1–67.

[50] Stekhoven DJ, Buhlmann P (2012). MissForest–Non-Parametric Missing Value Imputation for Mixed-Type Data. Bioinformatics.

[51] IBM Corp. IBM SPSS Statistics for Windows. Version 26.0. IBM Corp.; 2011.

[52] Chauncey HH, Muench ME, Kapur KK, Wayler AH (1984). The Effect of the Loss of Teeth on Diet and Nutrition. Int Dent J.

[53] Gunne H-SJ (1985). The Effect of Removable Partial Dentures on Mastication and Dietary Intake. Acta Odontol Scand.

[54] Gunne H-SJ, Wall A-K (1985). The Effect of New Complete Dentures on Mastication and Dietary Intake. Acta Odontol Scand.

[55] Akeel RF (1992). Masticatory Efficiency, a Literature Review. Saudi Dent J.

[56] Millwood J, Heath MR (2000). Food Choice by Older People: The Use of Semi-Structured Interviews with Open and Closed Questions. Gerodontology.

[57] Goiato MC, Ribeiro PDP, Garcia AR, dos Santos DM (2008). Complete Denture Masticatory Efficiency: A Literature Review. J Calif Dent Assoc.

[58] Van Der Bilt A, Burgers M, Van Kampen FMC, Cune MS (2010). Mandibular Implant-Supported Overdentures and Oral Function: Overdentures and Oral Function. Clin Oral Implant Res.

[59] Müller F (2014). Interventions for Edentate Elders - What Is the Evidence?. Gerodontology.

[60] Boven GC, Raghoebar GM, Vissink A, Meijer HJA (2015). Improving Masticatory Performance, Bite Force, Nutritional State and Patient’s Satisfaction with Implant Overdentures: A Systematic Review of the Literature. J Oral Rehabil.

[61] Takeshita H, Ikebe K, Gondo Y, Inagaki H, Masui Y, Inomata C, Mihara Y, Uota M, Matsuda K, Kamide K (2016). Association of Occlusal Force with Cognition in Independent Older Japanese People. JDR Clin Trans Res.

[62] Scheffels JF, Kräling H, Kalbe E, Kessler J (2018). Konversionen von kognitiven Screenings: Mini-Mental-Status-Test vs Montreal Cognitive Assessment vs DemTect. Nervenarzt.

[63] Kochhann R, Varela JS, de MacedoLisboa CS, Chaves MLF (2010). The Mini Mental State Examination: Review of Cutoff Points Adjusted for Schooling in a Large Southern Brazilian Sample. Dement Neuropsychol.

[64] O’Bryant SE, Humphreys JD, Smith GE, Ivnik RJ, Graff-Radford NR, Petersen RC, Lucas JA (2008). Detecting Dementia With the Mini-Mental State Examination in Highly Educated Individuals. Arch Neurol.

[65] Ciesielska N, Sokołowski R, Mazur E, Podhorecka M, Polak-Szabela A, Kędziora-Kornatowska K (2016). Is the Montreal Cognitive Assessment (MoCA) Test Better Suited than the Mini-Mental State Examination (MMSE) in Mild Cognitive Impairment (MCI) Detection among People Aged over 60?. Meta-Analysis Psychiatr Pol.

[66] Ikebe K, Gondo Y, Kamide K, Masui Y, Ishizaki T, Arai Y, Inagaki H, Nakagawa T, Kabayama M, Ryuno H (2018). Occlusal Force Is Correlated with Cognitive Function Directly as Well as Indirectly via Food Intake in Community-Dwelling Older Japanese: From the SONIC Study. PLoS One.

[67] Mlinarić A, Horvat M, Šupak Smolčić V (2017). Dealing with the Positive Publication Bias: Why You Should Really Publish Your Negative Results. Biochemia Medica.

[68] Lee YL, Lee BH, Lee SY (2019). Handgrip Strength in the Korean Population: Normative Data and Cutoff Values. Ann Geriatr Med Res.

[69] Yoo J-I, Choi H, Ha Y-C (2017). Mean Hand Grip Strength and Cut-off Value for Sarcopenia in Korean Adults Using KNHANES VI. J Korean Med Sci.

[70] Hahn P, Spies C, Unglaub F, Mühldorfer-Fodor M (2018). Die Messung der Griffkraft: Wertigkeit und Grenzen. Orthopäde.

[71] Račić M, Pavlović J, Ivković N (2019). Handgrip Strength Cut-Off Values for the Undernutrition Risk Screening among Elderly Men and Women in Bosnia and Herzegovina. J Aging Res.

[72] Su H, Sun X, Li F, Guo Q (2021). Association between Handgrip Strength and Cognition in a Chinese Population with Alzheimer’s Disease and Mild Cognitive Impairment. BMC Geriatr.

[73] Esteban-Cornejo I, Ho FK, Petermann-Rocha F, Lyall DM, Martinez-Gomez D, Cabanas-Sánchez V, Ortega FB, Hillman CH, Gill JMR, Quinn TJ (2022). Handgrip Strength and All-cause Dementia Incidence and Mortality: Findings from the UK Biobank Prospective Cohort Study. J Cachexia Sarcopenia Muscle.

[74] McGrath R, Vincent BM, Hackney KJ, Robinson-Lane SG, Downer B, Clark BC (2020). The Longitudinal Associations of Handgrip Strength and Cognitive Function in Aging Americans. J Am Med Dir Assoc.

[75] Lauretani F, Russo CR, Bandinelli S, Bartali B, Cavazzini C, Di Iorio A, Corsi AM, Rantanen T, Guralnik JM, Ferrucci L (1985). Age-Associated Changes in Skeletal Muscles and Their Effect on Mobility: An Operational Diagnosis of Sarcopenia. J Appl Physiol.

[76] Fried LP, Tangen CM, Walston J, Newman AB, Hirsch C, Gottdiener J, Seeman T, Tracy R, Kop WJ, Burke G (2001). Frailty in Older Adults: Evidence for a Phenotype. J Gerontol A Biol Sci Med Sci.

[77] Poyiadjis YM, Likeman PR (1984). Some Clinical Investigations of the Masticatory Performance of Complete Denture Wearers. J Dent.

[78] Anastassiadou V, Heath MR (2001). The Development of a Simple Objective Test of Mastication Suitable for Older People Using Chewing Gums. Gerodontology.

[79] Eberhard L, Schindler HJ, Hellmann D, Schmitter M, Rammelsberg P, Giannakopoulos NN (2012). Comparison of Particle-Size Distributions Determined by Optical Scanning and by Sieving in the Assessment of Masticatory Performance: MASTICATORY PERFORMANCE: VALIDATION OF A SCANNING METHOD. J Oral Rehabil.

[80] Verma TP, Kumathalli KI, Jain V, Kumar R (2017). Bite Force Recording Devices - A Review. J Clin Diagn Res.

[81] Trautwein S, Barisch-Fritz B, Scharpf A, Bossers W, Meinzer M, Steib S, Stein T, Bös K, Stahn A, Niessner C (2019). Recommendations for Assessing Motor Performance in Individuals with Dementia: Suggestions of an Expert Panel – a Qualitative Approach. Eur Rev Aging Phys Act.

[82] Cruz-Jentoft AJ, Baeyens JP, Bauer JM, Boirie Y, Cederholm T, Landi F, Martin FC, Michel J-P, Rolland Y, Schneider SM (2010). Sarcopenia: European Consensus on Definition and Diagnosis: Report of the European Working Group on Sarcopenia in Older People. Age Ageing.

[83] Chien M-Y, Huang T-Y, Wu Y-T (2008). Prevalence of Sarcopenia Estimated Using a Bioelectrical Impedance Analysis Prediction Equation in Community-Dwelling Elderly People in Taiwan: PREVALENCE OF SARCOPENIA IN TAIWAN. J Am Geriatr Soc.

[84] NIH Bioelectrical Impedance Analysis in Body Composition Measurement (1996). National Institutes of Health Technology Assessment Conference Statement. Am J Clin Nutr.

[85] Kyle UG, Genton L, Slosman DO, Pichard C (2001). Fat-Free and Fat Mass Percentiles in 5225 Healthy Subjects Aged 15 to 98 Years. Nutrition.

[86] Kyle UG, Genton L, Karsegard L, Slosman DO, Pichard C (2001). Single Prediction Equation for Bioelectrical Impedance Analysis in Adults Aged 20–94 Years. Nutrition.

[87] Roubenoff R, Baumgartner RN, Harris TB, Dallal GE, Hannan MT, Economos CD, Stauber PM, Wilson PWF, Kiel DP (1997). Application of Bioelectrical Impedance Analysis to Elderly Populations. J Gerontol A Biol Sci Med Sci.

[88] Working Group on Functional Outcome Measures for Clinical Trials (2008). Functional Outcomes for Clinical Trials in Frail Older Persons: Time To Be Moving: Working Group on Functional Outcome Measures for Clinical Trials. The Journals of Gerontology Series A. J Gerontol A Biol Sci Med Sci.

[89] van de Rijt LJM, Feast AR, Vickerstaff V, Sampson EL, Lobbezoo F (2021). Oral Function and Its Association with Nutrition and Quality of Life in Nursing Home Residents with and without Dementia: A Cross-Sectional Study. Gerodontology.

